# Estimation of stride-by-stride spatial gait parameters using inertial measurement unit attached to the shank with inverted pendulum model

**DOI:** 10.1038/s41598-021-81009-w

**Published:** 2021-01-14

**Authors:** Yufeng Mao, Taiki Ogata, Hiroki Ora, Naoto Tanaka, Yoshihiro Miyake

**Affiliations:** 1 Department of Computer Science, Tokyo Institute of Technology, Kanagawa, 226-8503 Japan; 2 Department of Systems and Control Engineering, Tokyo Institute of Technology, Kanagawa, 226-8503 Japan

**Keywords:** Human behaviour, Engineering, Physiology

## Abstract

Inertial measurement unit (IMU)-based gait analysis systems have become popular in clinical environments because of their low cost and quantitative measurement capability. When a shank is selected as the IMU mounting position, an inverted pendulum model (IPM) can accurately estimate its spatial gait parameters. However, the stride-by-stride estimation of gait parameters using one IMU on each shank and the IPMs has not been validated. This study validated a spatial gait parameter estimation method using a shank-based IMU system. Spatial parameters were estimated via the double integration of the linear acceleration transformed by the IMU orientation information. To reduce the integral drift error, an IPM, applied with a linear error model, was introduced at the mid-stance to estimate the update velocity. the gait data of 16 healthy participants that walked normally and slowly were used. The results were validated by comparison with those extracted from an optical motion-capture system; the results showed strong correlation ($r>0.9$) and good agreement with the gait metrics (stride length, stride velocity, and shank vertical displacement). In addition, the biases of the stride length and stride velocity extracted using the motion capture system were smaller in the IPM than those in the previous method using the zero-velocity-update. The error variabilities of the gait metrics were smaller in the IPM than those in the previous method. These results indicated that the reconstructed shank trajectory achieved a greater accuracy and precision than that of previous methods. This was attributed to the IPM, which demonstrates that shank-based IMU systems with IPMs can accurately reflect many spatial gait parameters including stride velocity.

## Introduction

In recent years, many studies have focused on the development of an inertial measurement unit (IMU) equipped with an accelerometer, gyroscope sensor, and magnetometer for a gait analysis system that can provide quantitative gait parameters such as stride length, velocity, gait cycle. Compared to the golden standard for gait analysis—a motion capture system and instrumented walkways—the IMU-based system is cost effective, lightweight, and versatile, which are suitable characteristics for clinical and residential applications^1^. In particular, the inverted pendulum model (IPM) is considered useful for estimating spatial gait parameters such as stride length from acceleration and angular velocities measured by the IMU^2,3^. However, the step-by-step accuracy of the spatial parameters estimated using the IMU data and IPM model has not been validated thus far.

Kinematics information in the gait cycle is a component of gait considered in clinical gait analyses^4^. For example, variability in stride length can be used to assess the progression of Parkinson’s disease (PD)^5^, and stride velocity can be used to predict the risk of adverse events in the elderly^6^. To estimate spatial gait parameters from acceleration and angular velocities measured by the IMU, the previous studies implemented a segmentation algorithm^7–10^. Integral computations for spatial parameter estimation are reset at each segmentation point, which reduces errors caused by the measurement noise. The implementation details of these algorithms depend on the attachment position of the IMU because different positions produce different signal characteristics and different assumptions need to be considered to improve estimation accuracy. A wide selection of attachment positions for the IMUs involved bilateral shanks^7,11^, bilateral insteps^12–15^, and bilateral heels^16,17^. In terms of ensuring attachment stability, the shank position seems to be a good choice because the soft tissue of the lower shank has less movement than that at the other positions^18^. Further, when the IMU is fixed to the heel or instep position on footwear, the moving artifact of the footwear affects the estimation accuracy. However, compared to the heel or instep attachment positions closer to the ground, the shank position has room for improvement in terms of estimating spatial parameters such as stride length and stride velocity. The shank-based method can be used to obtain reliable gait parameters and visualise the 3D-trajectory of each stride^19,20^. In a previous study, zero-velocity-update (ZUPT) was introduced, which re-initialises the integrated velocity to zero at the segmentation point (mid-stance). However, the shank possesses a small velocity at the mid-stance^2^, which results in the error being included in the estimated spatial gait parameters. Instead of assuming the velocity at the mid-stance to be zero, the inverted pendulum model (IPM)^2,3^, which considers the IMU movement at the mid-stance as a circular motion in the sagittal plane and estimates the velocity using angular velocity has the potential to provide a more accurate result. In fact, Wu et al. showed that the total error of the walking distance estimated using shank-mounted IMUs and an IPM was smaller than that estimated using the same IMUs and a ZUPT^19^.

However, it remains unclear if stride-by-stride estimation using shank-mounted IMUs and the IPM is superior to that using ZUPT. As mentioned above, some variability in the spatial gait parameters is important for the gait assessment of people with gait disorders, and to investigate such variability in gait parameters, stride-by-stride estimation of gait parameters is necessary. This study aimed to validate the stride-by-stride estimation method for spatial gait parameters using shank-mounted IMUs and the IPM. In addition, we investigated whether the stride-by-stride estimation of the spatial gait parameters using the IPM was superior to that using the ZUPT. A modified IPM that considered the posture of the IMU for shank trajectory estimation was implemented. For evaluating the proposed method, we performed a concurrent validation experiment using an optical motion capture system with a high spatial resolution. Next, we compared the proposed method to a ZUPT method to investigate whether the IPM improved the accuracy of the stride-by-stride estimation. We used a previously reported method^20^ as the ZUPT method because this method had been evaluated for stride-by-stride spatial gait parameters.

## Results

All 16 participants completed both tasks; the data for the normal speed task of one of the participants could not be analysed because of synchronisation issues (the motion capture system did not capture the stamp event), and therefore, it was excluded. The motion capture system suffered from loss of data because of the reflective markers; these invalid data were excluded, and a total of 695 strides were extracted from the motion capture system, with 283 strides for the normal speed task and 412 strides for the slower speed task.

**Figure 1:**
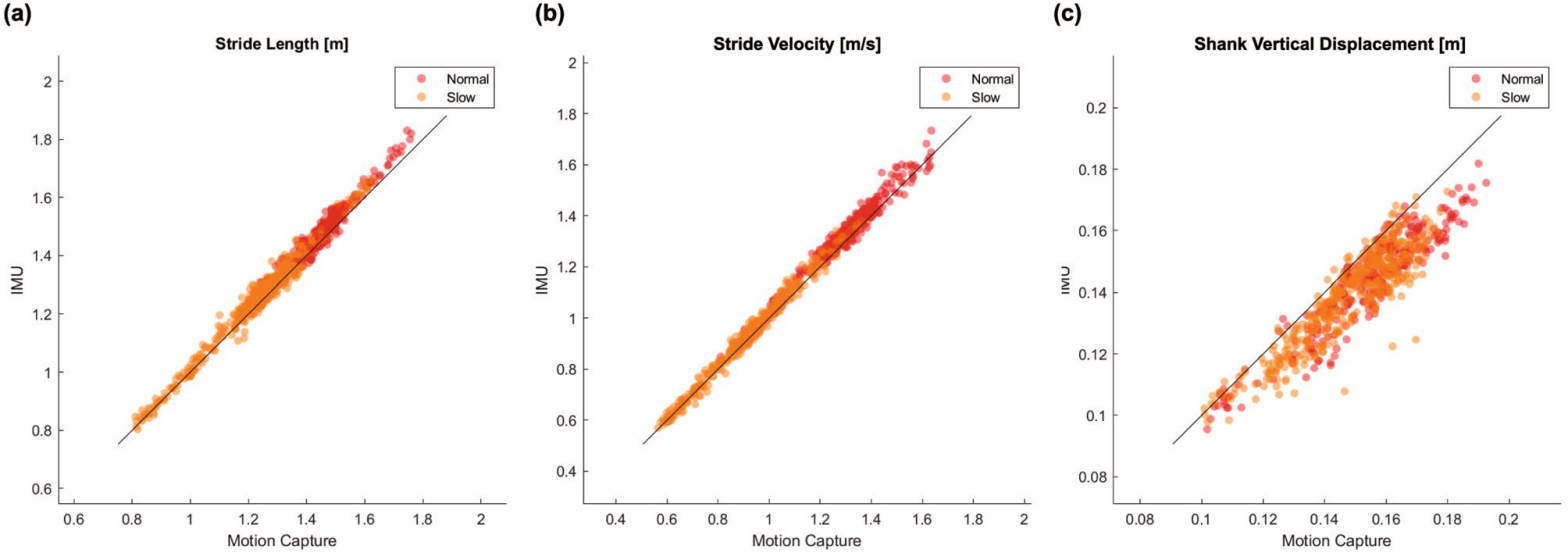
Scatter plots of (**a**) stride length, (**b**) stride velocity, and (**c**) shank vertical displacement extracted from the proposed method and motion capture system. Each dot indicates the value in one stride. The black line shows the best fit line.

First, we validated the proposed method compared to the results from the motion capture system. Figure 1 shows scatter plots for the stride length, stride velocity, and shank vertical displacement between the data extracted from the proposed method and those obtained from the motion capture system. The stride length and velocity decreased when the participants walked slowly. Pearson’s correlation coefficient and the error distribution are summarised in Table 1. All parameters achieve high correlation with Pearson correlation coefficient $r > 0.90.$ Thus, the proposed method estimated the parameters appropriately.

**Figure 2:**
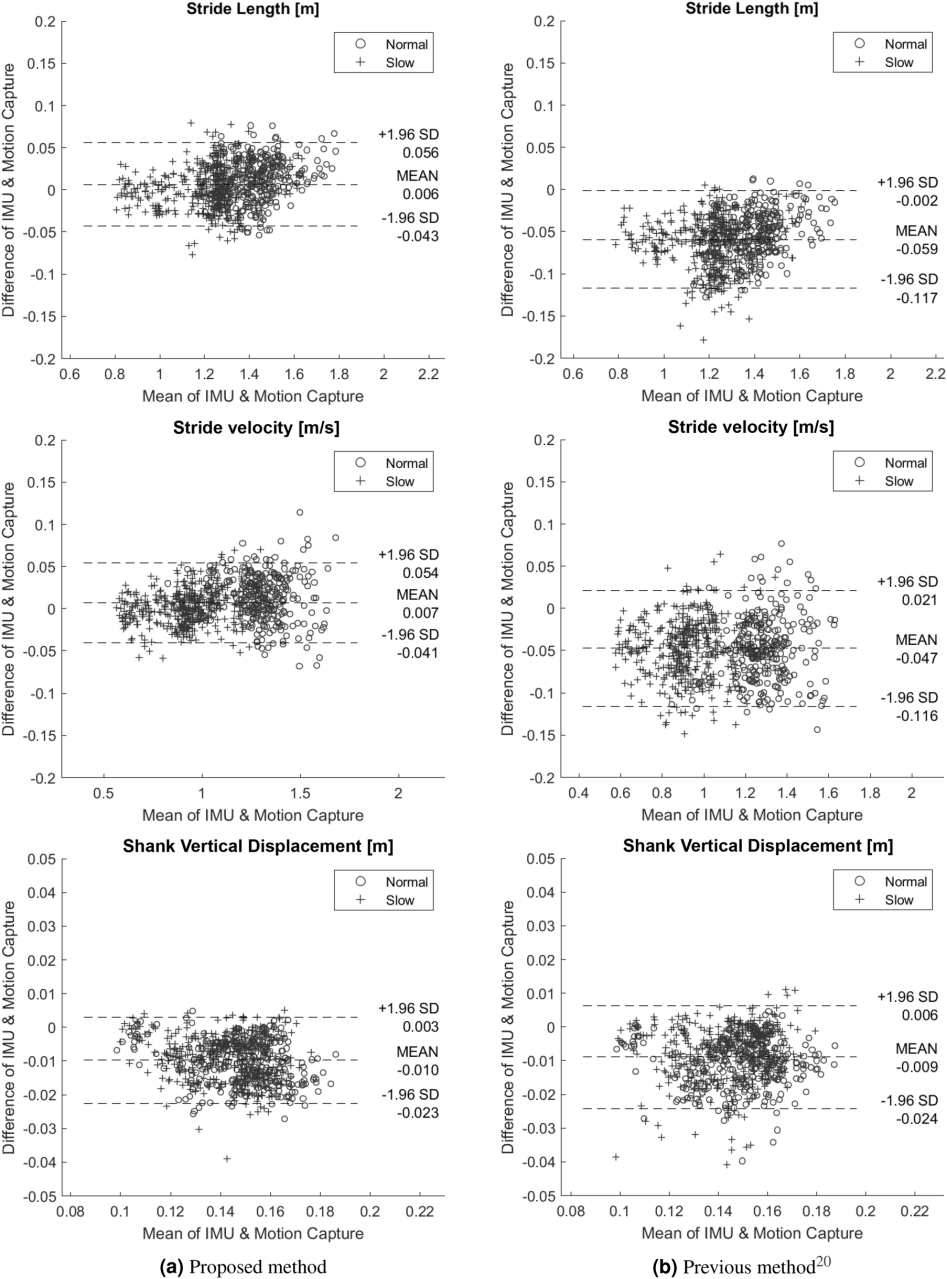
Bland–Altman plot for stride length, stride velocity, and shank vertical displacement estimated by (**a**) the proposed method and (**b**) the previous method^20^ for 283 strides under the normal speed task ($\circ$) and 412 strides under the slower speed task (+). The Bland–Altman plot provides information about the mean difference, which indicates the bias between two systems, and the 95% confidence interval, which is known as the limit of agreement (LOA) that shows the difference between the values measured by two systems for most individuals^27^.

Second, we investigated whether the proposed method estimated the parameters more accurately than the previous method. The Bland–Altman plots for the parameters estimated using the proposed method and a previous method are shown in Fig. 2. The biases of stride length, stride velocity, and shank vertical displacement for the proposed and previous methods were 0.006 m and $-0.059$ m, 0.007 m/s and $-0.047$ m/s, and $-0.010$ m and $-0.009$  m, respectively. The stride length and stride velocity estimated using the proposed method achieved a very small bias compared with those estimated using the previous method. The 95% confidence intervals of stride length, stride velocity, and shank vertical displacement for the proposed and previous methods were 0.099 m and 0.115 m, 0.095 m/s and 0.137 m/s, and 0.026 m and 0.030 m, respectively. Thus, the error variability for all parameters estimated using the proposed method was smaller than that using the previous method.

**Table 1:** Pearson’s correlation coefficient (*r*), the mean (SD) of error (*E*), absolute error (|*E*|), and relative absolute errors ($|E|\%$) for the gait parameters extracted from the proposed method and motion capture system.

Parameters	*r*	*E*	|*E*|	$|E|\%$
**Stride length (m)**				
Normal	0.98	0.012 (0.026)	0.024 (0.016)	1.7% (1.1%)
Slow	0.99	0.002 (0.025)	0.020 (0.015)	1.6% (1.2%)
Overall	1.00	0.007 (0.025)	0.020 (0.016)	1.9% (1.4%)
**Shank vertical displacement (m)**				
Normal	0.93	− 0.011 (0.007)	0.011 (0.006)	6.9% (3.9%)
Slow	0.91	− 0.009 (0.007)	0.009 (0.006)	6.3% (4.0%)
Overall	0.92	− 0.010 (0.007)	0.010 (0.006)	6.5% (4.0%)
**Stride velocity (m/s)**				
Normal	0.98	0.014 (0.028)	0.026 (0.018)	2.0% (1.4%)
Slow	0.99	0.002 (0.021)	0.016 (0.013)	1.8% (1.4%)
Overall	1.00	0.007 (0.025)	0.020 (0.016)	1.9% (1.4%)

## Discussion

This study aimed to validate stride-by-stride gait estimation using IMUs attached to the shank and an IPM. The parameters were extracted from the walking data of 16 participants, and they were compared with those obtained using a motion capture system as reference. The results showed high correlation and good agreement between the two systems for stride length, stride velocity, and shank vertical displacement, which verifies the technical effectiveness of the proposed method.

In terms of the Bland–Altman analysis, the proposed method achieved a $-0.044$ to 0.057 m limit of agreement for the stride length, which is comparable to that achieved using the heel- or instep-based method^14,15,17,21^. The relative absolute error of the shank vertical displacement is slightly worse than other parameters with an overall value of 6.5% (Table 1). When comparing the spatial parameters in the forward (stride length) and vertical directions (shank vertical displacement), the magnitude of the error tended to be different. The value of movement in the vertical direction was smaller than that in the forward direction, and therefore, the shank vertical displacement would be susceptible to noise. However, the shank vertical displacement still achieved good agreement with a mean of -0.010 m and the limit of agreement of $-0.023$  m and 0.003 m.

Compared to the previous method^20^ as shown in Fig. 2, the proposed method achieved a considerably smaller bias with a stride length of 0.005 m and a stride velocity of 0.007 m/s. This is because the modified IPM used in this study compensates the velocity estimation error at each segmentation point. Further, this result demonstrated that the IPM achieves good performance in terms of 3D-trajectory estimation. The previous IPM required an event assumption that the shank tilt angle in the sagittal plane is zero; however, this was difficult to guarantee^22^. The the proposed method combines orientation estimation that computes the update velocity in three dimensions and does not require an event assumption. The estimated shank vertical displacement from both methods still have a close bias, which indicates that the vertical direction has a very small velocity at the segmentation point and does not gain considerable benefit from the IPM. The bias of the shank vertical displacement that appears in both methods can be affected by IMU calibration where the linear acceleration in the vertical direction is calculated by subtracting the gravity component^23,24^.

The above results indicates that the IPM contributes to spatial gait parameter estimation with sufficient accuracy compared to the motion-capture system. The limitation of the current study is that it does not use patient data or elderly data. However, although measurement targets can walk such that the sole of their foot contacts the ground and the feasibility of the pendulum model for abnormal gait assessed in a prior research^2^ indicates that the proposed method can be applied to patients or the elderly, this still needs to be proved. In particular, the IPM may be strict in estimating the gait parameters of people with severe gait disorders. For example, the IPM may work well when people limp or shuffle because their gait trajectories would not fit to the pendulum model. Future research needs to address the gait events detection problem for abnormal gait because it is the prerequisite for most segmentation-algorithm-based gait trajectory estimation methods. However, the IPM would be useful in distinguishing people with slight gait disorders from healthy people. For example, some studies have attempted to classify early PD patients and healthy elders using gait parameters to develop an early diagnosis method for PD patients^25,26^. Because the gait of early PD patients is similar to that of healthy people, it is necessary to estimate the walking trajectory as accurately as possible. Therefore, the IPM would work well to classify early PD patients and healthy elders.

For data processing, we did not use high- or low-pass filters for the IMU data because we initially considered that the proposed method could almost eliminate the drift. In fact, the proposed method showed high accuracy in trajectory estimation. However, such filters would theoretically improve the estimation accuracy. Some studies developed a filtering method for IMU data in gait trajectory estimation^23,24^. In the future, such filters would be applied or added to the proposed method.

Although we could not discuss gender-related difference for the limitation number of the participants, it is possible to increase the accuracy of trajectory estimation using gender differences in the gait. In fact, some studies investigated gender differences in the gait^28–30^. For example, women’s foot progression angle tends to be more internally rotated than those of men in the stance phase^28^. This difference can affect the trajectory and estimation accuracy of the trajectory.

## Conclusions

In this study, an IMU-based stride-by-stride gait analysis method was validated with an optical motion capture system. The method explored the possibility of using the IPM to analyse the shank trajectory that estimates the stride length, stride velocity, and shank vertical displacement. The accuracy of each parameter estimated by the IPM method was compared with that estimated using a previous method based on the ZUPT. Using the proposed method, the average error of the extracted stride length achieved a small bias of 0.006 m. Overall, this study is a step toward the development of a shank-based gait analysis system that will serve as a convenient objective measurement tool for future clinical diagnoses.

## Methods

### System setups

**Figure 3:**
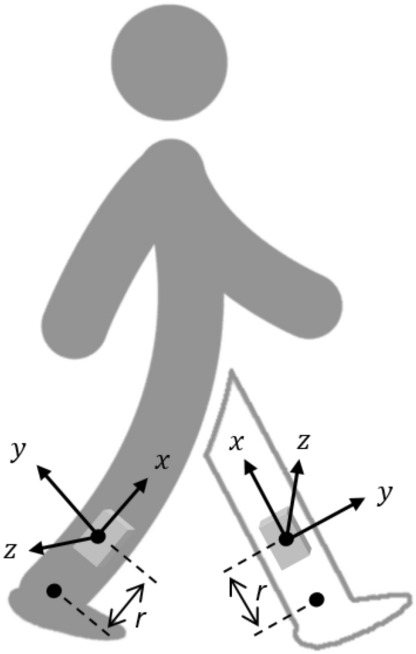
Configuration of the IMUs. Two IMUs are attached to the shank position right above the malleolus at a distance of *r*. The axes *x*, *y*, *z* are the coordinate system of the IMU, where the *z*-axis is perpendicular to the sagittal plane formed by the *y* and *z*-axes.

In this study, two IMUs (accelerometer and gyroscope) were mounted on the shanks on both sides, immediately above the malleolus at a distance of *r*. The attachment position and coordinate system of the IMUs are shown in Fig. 3. The x, y, and z axes represented inferior/superior, posterior/anterior, and medial/lateral directions, respectively.

### Gait event detection and data segmentation via angular velocity

**Figure 4:**
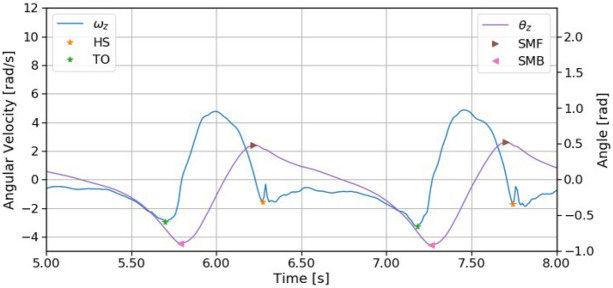
Samples of angular velocity, angle in the sagittal plane, and gait events. The heel-strike (HS) and toe-off (TO) events were defected from the angular velocity.

Heel-strike (HS) and toe-off (TO) events were first detected based on the angular velocity in the sagittal plane $\omega _{z}$^7^ (Fig. 4). The search regions for HS and TO events were defined based on the shank tilt angle in the sagittal plane $\theta _z$^20^ calculated using the integration of $\omega _z$. Then, each HS was defined as the first peak that appears after the local maximum of $\theta _z$ (shank max forward; SMF) and each TO was defined as the minimum of $\omega _z$ that appears before the local minimum of $\theta _z$ (shank max backward; SMB). After the HS and TO in each gait cycle were detected, the data were segmented by the mid-stance (MS), which is defined as the maximum point of $\omega _z$ between HS and TO^11^.

### Orientation estimation from acceleration and angular velocity

The quaternion system was introduced to describe orientation information in this study. We use superscripts to represent the coordinate frame wherein a variable is located and the rotation direction of a quaternion. Superscripts *S* and *E* represent the IMU coordinate frame and the laboratory coordinate frame, respectively. Further, we use (*k*) to represent the *k*th sample of an instantaneous variable and (*i*) to represent a variable in the *i*th gait cycle. For example, ${\varvec{q}}^{SE}(k)$ represents the instantaneous quaternion that can convert a vector from frame *S* to frame *E*. In addition, *ms*(*i*) is the mid-stance event in the *i*th gait cycle.

At each *ms*(*i*), we assumed that the IMU is reset and the accelerometer only detects gravity. Therefore, the rotation quaternion ${\varvec{q}}^{SE}(k)$ at *ms*(*i*) is computed using the accelerometer output ${\varvec{a}}^S(k)$ and gravity $${\varvec{g}}^{E} = \begin{bmatrix} {1}&{0}&{0} \end{bmatrix}^T$$ as

1 $$\begin{aligned} {\varvec{q}}^{SE}(k)= \begin{bmatrix} {\cos {\frac{\theta (k)}{2}} }&{\varvec{n}(k) \sin { \frac{\theta (k)}{2} }} \end{bmatrix} ,~ k=ms(i) \end{aligned}$$

with

2 $$\begin{aligned} \cos \frac{\theta (k)}{2}= & {} \sqrt{\frac{1}{2} \left( 1 + \frac{{\varvec{a}}^S(k)}{\left\Vert \varvec{a}^S(k)\right\Vert } \cdot {\varvec{g}}^{E} \right) } ,~ k=ms(i) \end{aligned}$$

3 $$\begin{aligned} \sin \frac{\theta (k)}{2}= & {} \sqrt{\frac{1}{2} \left( 1 - \frac{\varvec{a}^S(k)}{\left\Vert \varvec{a}^S(k)\right\Vert } \cdot {\varvec{g}}^{E} \right) } ,~ k=ms(i) \end{aligned}$$

4 $$\begin{aligned} \varvec{n}(k)= & {} \frac{\varvec{a}^S(k)}{\left\Vert \varvec{a}^S(k)\right\Vert } \times \varvec{g}^E,~ k=ms(i) \end{aligned}$$

where ∥ ∥ represents the L2-norm, $\cdot$ is the dot product, and $\times$ is the cross product.

The remaining rotation quaternions in each segment were computed via the integration of angular velocity $\varvec{\omega }^S(k)$ for each sample as

5 $$\begin{aligned} \varvec{q}^{SE}(k)= \frac{\varvec{q}^{SE}(k-1) + { \dot{\varvec{q}}^{SE}(k)\Delta t }}{\left\Vert \varvec{q}^{SE}(k-1) + { \dot{\varvec{q}}^{SE}(k)\Delta t }\right\Vert } ,~ k \in (ms(i), ms(i+1)) \end{aligned}$$

with

6 $$\begin{aligned} \dot{\varvec{q}}^{SE}(k)= \frac{1}{2} \varvec{q}^{SE}(k-1) \otimes \begin{bmatrix} {0}&{\varvec{\omega }^S(k)} \end{bmatrix} ,~ k \in (ms(i), ms(i+1)) \end{aligned}$$

where $\otimes$ denotes the quaternion product and $\Delta t$ represents the sampling interval.

Using $\varvec{q}^{SE}(k),$ we can transform the measured acceleration into the laboratory coordinate frame as

7 $$\begin{aligned} \begin{bmatrix} { 0 }&{ \varvec{a}^E(k) } \end{bmatrix} = \varvec{q}^{SE}(k)\otimes \begin{bmatrix} {0}&{\varvec{a}^S(k)} \end{bmatrix} \otimes {(\varvec{q}^{SE}(k))^{*}} \end{aligned}$$

where $(\varvec{q}^{SE}(k))^{*}$ is the conjugate of $\varvec{q}^{SE}(k).$

Finally, the linear acceleration ${\tilde{\varvec{a}}}^E(k)$ is obtained by removing the gravitational component from $\varvec{a}^E(k)$.

8 $$\begin{aligned} {\tilde{\varvec{a}}}^E(k) = \varvec{a}^E(k) - {\varvec{g}}^{E} \end{aligned}$$

### Velocity estimation and drift removal via inverted pendulum model

After linear acceleration is determined, the velocity $\varvec{v}^E(k)$ is computed using the trapezoidal integral with the sampling interval as

9 $$\begin{aligned} \varvec{v}^E(k) = \varvec{v}^E(k-1) + \frac{ {\tilde{\varvec{a}}}^E(k) + {\tilde{\varvec{a}}}^E(k-1) }{ 2 } \Delta t \end{aligned}$$

**Figure 5:**
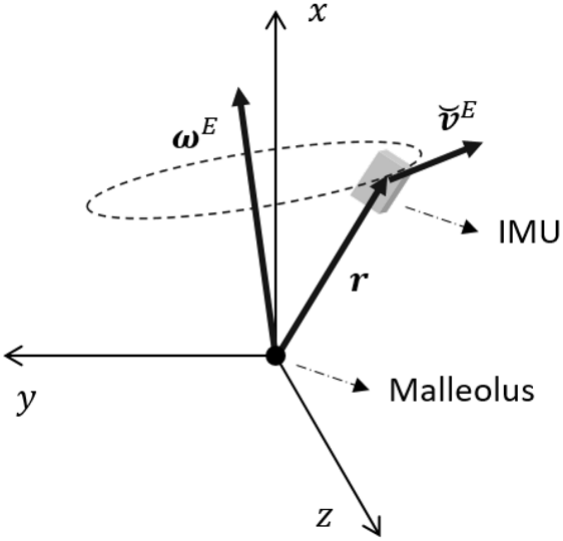
Motion of the IMU at the mid-stance can be modelled as a rotational motion in a three-dimensional space. The position vector $\varvec{r}$ and angular velocity in laboratory coordinate frame ${\omega }^{E}$ were computed from the estimated orientation of IMU. Thus, the update velocity ${\breve{\varvec{v}}}^{E}$ can be recovered as the tangential velocity associated with $\varvec{r}$ and ${\omega }^{E}.$

However, this integral result contains significant drift. This drift occurs from the accumulation of sensor errors such as bias in the gyroscope and jitter in the accelerometer. To solve this problem, a modified IPM was introduced in this phase. At each *ms*(*i*), we assume the movement of the shank to be rotational motion with the malleolus as a fulcrum (Fig. 5). Thus, the velocity at *ms*(*i*) can be calculated as the cross product between the angular velocity in the laboratory coordinate frame $\varvec{\omega }^E(k)$ and the position $\varvec{r}(k)$ of the IMU

10 $$\begin{aligned} \breve{\varvec{v}}^E(k) = \varvec{\omega }^E(k) \times \varvec{r}(k) ,~ k = ms(i) \end{aligned}$$

with

11 $$\begin{aligned} \varvec{\omega }^E(k) = \varvec{q}^{SE}(k)\otimes \varvec{\omega }^S(k) \otimes (\varvec{q}^{SE}(k))^{*}.\end{aligned}$$

We assume that $\varvec{a}^S(k)$ contains the gravitational component only at *ms*(*i*), and therefore, $\varvec{r}(k)$ is defined as the vector in the $\varvec{\varvec{a}}^S(k)$ direction with a magnitude of the distance between the IMU and the malleolus *r* as

12 $$\begin{aligned} \varvec{r}(k) = r \frac{\varvec{a}^S(k)}{\left\Vert \varvec{a}^S(k)\right\Vert } ,~ k = ms(i). \end{aligned}$$

If the IMU sensor is attached at a certain distance from the ankle, *r* is known in advance and there is no need to measure it each time.

Then, we model the drift error $\varvec{e}(k)$ as a linear variation^31^ that occurs over time with a constant slope and intercept in each segment as

13 $$\begin{aligned} \varvec{e}(k) = \varvec{\alpha }(i) (k - ms(i)) + \varvec{\beta }(i) ,~ k \in [ms(i), ms(i+1)] \end{aligned}$$

where $\varvec{\alpha }(i)$ and $\varvec{\beta }(i)$ are the slope and the intercept of the error model in the *i*th gait cycle, respectively.

Using the velocity $\breve{\varvec{v}}^E(k)$ estimated from the IPM, $\varvec{\alpha }(i)$ and $\varvec{\beta }(i)$ are computed as

14 $$\begin{aligned} \varvec{\alpha }(i)= & {} \frac{ \varvec{v}^E(ms(i+1)) - \breve{\varvec{v}}^E(ms(i+1)) - \varvec{v}^E(ms(i)) + \breve{\varvec{v}}^E(ms(i)) }{ ms(i+1) - ms(i) } \end{aligned}$$

15 $$\begin{aligned} \varvec{\beta }(i)=\, & {} \varvec{v}^E(ms(i)) - \breve{\varvec{v}}^E(ms(i)) \end{aligned}$$

Finally, the drift error is removed from velocity $\varvec{v}^E(k)$ by subtracting the modelled error, and the correction result ${\tilde{\varvec{v}}}^E(k)$ is obtained as

16 $$\begin{aligned} {\tilde{\varvec{v}}}^E(k) = \varvec{v}^E(k) - \varvec{\alpha }(i) (k - ms(i)) - \varvec{\beta }(i) ,~ k \in [ms(i), ms(i+1)] \end{aligned}$$

### Trajectory estimation and coordinate frame transformation

First, the trajectory $\varvec{p}^E(k)$ is estimated by the direct integration of the corrected velocity ${\tilde{\varvec{v}}}^E(k)$ via a trapezoidal rule first as

17 $$\begin{aligned} \varvec{p}^E(k) = \varvec{p}^E(k-1) + \frac{{\tilde{\varvec{v}}}^E(k) + {\tilde{\varvec{v}}}^E(k-1) }{2} \Delta t \end{aligned}$$

Then, a new coordinate frame *P* is introduced for visualising the stride trajectory and computing the spatial parameters. In frame *P*, the *y* axis direction is aligned to the stride forward direction and the *x* axis is aligned along the vertical direction. The rotation matrix $\varvec{R}^{EP}(i)$ between the frames *P* and *E* is computed by solving the equation

18 $$\begin{aligned} \varvec{I}_3 = \varvec{R}^{EP}(i) \cdot \varvec{P}^E(i) \end{aligned}$$

where

19 $$\begin{aligned} \varvec{I}_3 = \begin{bmatrix} 1 &{}\quad 0 &{}\quad 0 \\ 0 &{}\quad 1 &{}\quad 0 \\ 0 &{}\quad 0 &{}\quad 1 \end{bmatrix} \end{aligned}$$

and each column of matrix $$ \varvec{P}^E(i) = \begin{bmatrix} {\varvec{x}^E(i)}&{\varvec{y}^E(i)}&{\varvec{z}^E(i)} \end{bmatrix} $$ is defined as

20 $$\begin{aligned} \varvec{y}^E(i)=\, & {} \frac{\varvec{p}^E(ms(i+1)) - \varvec{p}^E(ms(i))}{\left\Vert \varvec{p}^E(ms(i+1)) - \varvec{p}^E(ms(i))\right\Vert } \end{aligned}$$

21 $$\begin{aligned} \varvec{z}^E(i)=\, & {} \frac{\begin{bmatrix} {1}&{0}&{0} \end{bmatrix}^T \times \varvec{y}^E(i) }{\left\Vert \begin{bmatrix} {1}&{0}&{0} \end{bmatrix}^T \times \varvec{y}^E(i) \right\Vert } \end{aligned}$$

22 $$\begin{aligned} \varvec{x}^E(i)=\, & {} \varvec{y}^E(i) \times \varvec{z}^E(i) \end{aligned}$$

The final estimate trajectory $\varvec{p}^P(k)$ in each gait cycle is transformed using the rotation matrix applied to $\varvec{p}^E(k)$:

23 $$\begin{aligned} \varvec{p}^P(k) = \varvec{R}^{EP}(i) \cdot \varvec{p}^E(k) ,~ k \in [ms(i), ms(i+1)) \end{aligned}$$

**Figure 6:**
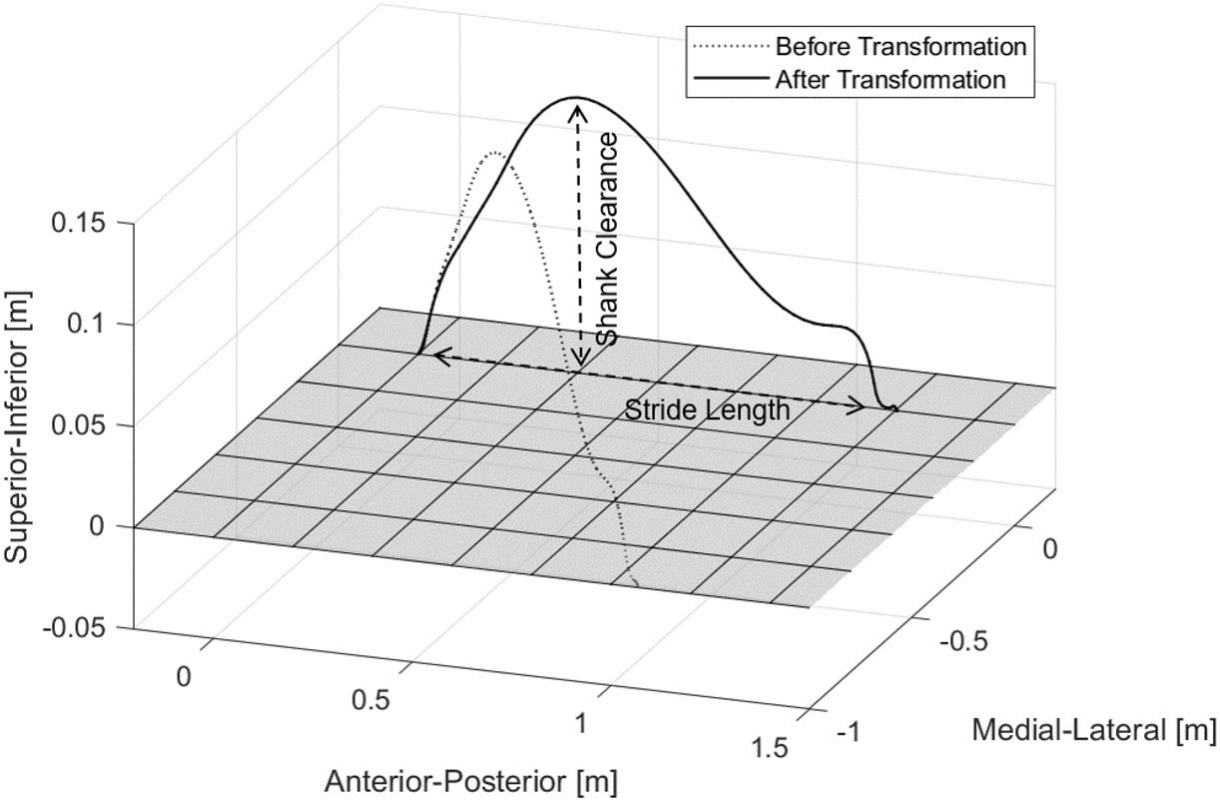
Typical ankle trajectory before (in frame *E*) and after transformation (in frame *P*). This transformation allows visualising the trajectory and computing the spatial gait parameters. Stride length is defined as the displacement in the anterior-posterior direction; shank vertical displacement is defined as the maximum displacement in the superior-inferior direction.

The example of the stride before and after the transformation is shown in Fig. 6.

### Spatial gait parameter computation

The definitions of stride length and shank vertical displacement are shown in Fig. 6. Stride length during one gait cycle is computed as the displacement in the *y* direction $p_y^P(k)$ between two successive mid-stance events.

24 $$\begin{aligned} \text {Stride length}(i) = p_y^P(ms(i+1)) -p_y^P(ms(i)) \end{aligned}$$

Shank vertical displacement is calculated as the maximum displacement value in the *x* direction $p_x^P(k)$.

25 $$\begin{aligned} \text {Shank vertical displacement}(i) = {\text {max}} ~ p_x^P(k) ,~ k \in (ms(i), ms(i+1)) \end{aligned}$$

Stride velocity is defined as the division of the stride length and stride duration.

26 $$\begin{aligned} \text {Stride velocity}(i) = \frac{\text {Stride length}(i)}{\text {Stride duration}(i)} \end{aligned}$$

with

27 $$\begin{aligned} \text {Stride duration}(i) = hs(i+1) - hs(i) \end{aligned}$$

## Evaluation experiment

In this study, six females and ten males (age: $23 \pm 2$; height: $164 \pm 7\;{\hbox {cm}}$) participated in the concurrent validation experiment. This experiment was conducted in accordance with the Declaration of Helsinki and approved by the ethics committee of the Tokyo Institute of Technology. Written informed consent was obtained from all participants.

The participants completed a $4 \times 10\;{\hbox {m}}$ walking tasks at two different self-selected walking speeds: normal and a slower speed, in a fixed order. In the future, we assume elders or patients with gait disorder such as PD patients as the target for our system. Aging and disorders decrease people’s gait velocity^32,33^, and therefore, we employed the slow walking condition. The participants conducted one trial under each condition. Two IMUs (TSND121, ATR-Promotions, Japan) with features of an accelerometer ($\pm 8\;{\hbox {g}}$) and a gyroscope ($\pm 1000 ^{\circ }/{\hbox {s}}$) were used to implement the proposed method. The size of the TSND121 is $37\;{\hbox {mm}} \times 46\;{\hbox {mm}} \times 12\;{\hbox {mm}}$ and the weight is approximately $22\;{\hbox {g}}$. Each IMU was placed into a housing pocket with an elastic band, and it was attached to the shank in the position $0.03\;{\hbox {cm}}$ above the malleolus.

A motion capture system with 12 cameras (VENUS3D, NOBBYTECH, Japan) and optical motion capture software (Motive:Tracker, NaturalPoint, Inc.) was used as the reference system. The motion capture system was calibrated well to ensure that the overall displacement error was under $1\;{\hbox {mm}}$. The sampling frequencies were set to $100\;{\hbox {Hz}}$ for both the IMU and the motion capture system. Before the start of the experiment, participants were asked to stamp their left and right foot once to synchronise events between the IMUs and the motion-capture system. We used Python (Python Software Foundation) for data processing and analysis.

For the statistical analysis, we compute the difference in the stride-by-stride gait parameter results extracted using the IMU and the motion-capture system as the error for the proposed method. We computed the mean and standard deviation of the error, the absolute error, and the relative absolute error for each parameter. Further, Bland–Altman analysis^34^ was introduced to assess the agreement between the IMU and the motion-capture measurement system for the previous^20^ and proposed methods.

One participants’ data in normal speed was excluded because the motion capture data cannot be synchronized with the IMU data. Finally, 15 normal speed data and 16 slower speed data were analyzed. After removing invalid data resulting from the reflective markers loss in the invisible area, a total of 722 strides were extracted from the motion capture system, with 289 strides (19.27 strides per one participant) in the normal speed task and 433 strides (27.06 strides per one participant) from the slower speed task. The number of strides analyzed is not less than that measured in previous studies^13,14,20^. All strides can be found in the IMU estimation results without missing detection. Thus, we analyzed these data to validate the proposed method.
